# Two-pore channels and disease^☆^

**DOI:** 10.1016/j.bbamcr.2018.05.004

**Published:** 2018-11

**Authors:** Sandip Patel, Bethan S. Kilpatrick

**Affiliations:** Department of Cell and Developmental Biology, University College London, Gower Street, London WC1E 6BT, United Kingdom

**Keywords:** TPCN1, TPCN2, NAADP, Ca^2+^, Lysosomes

## Abstract

Two-pore channels (TPCs) are Ca^2+^-permeable endo-lysosomal ion channels subject to multi-modal regulation. They mediate their physiological effects through releasing Ca^2+^ from acidic organelles in response to cues such as the second messenger, NAADP. Here, we review emerging evidence linking TPCs to disease. We discuss how perturbing both local and global Ca^2+^ changes mediated by TPCs through chemical and/or molecular manipulations can induce or reverse disease phenotypes. We cover evidence from models of Parkinson's disease, non-alcoholic fatty liver disease, Ebola infection, cancer, cardiac dysfunction and diabetes. A need for more drugs targeting TPCs is identified.

## 1 Introduction

Two-pore channels (TPCs) are ancient members of the voltage-gated ion channel superfamily [1]. Structurally, they are dimers with each subunit harbouring two similar ion channel domains connected by a cytoplasmic linker and each possessing six transmembrane helicies, [[2], [3], [4], [5]]. This duplicated domain architecture identifies TPCs as likely intermediates in the evolutionary transition from tetrameric one-domain channels (such as TRP channels) and monomeric four domain channels (such as voltage-gated Ca^2+^ channels) [6]. TPCs are unusual in that they reside on acidic organelles such as lysosomes [7,8] or the equivalent vacuoles in plants [9]. This is achieved through specific targeting motifs in their N-termini [10,11].

TPCs are thought to be non-selective cation channels permeable to Ca^2+^ [1]. They were originally functionally characterised as the long sought targets for the Ca^2+^ mobilizing messenger NAADP [7,8,12,13]. This second messenger, discovered in sea urchin eggs [14], has an established role in releasing Ca^2+^ from so called acidic Ca^2+^ stores [15,16]. NAADP regulates numerous functions from fertilisation in echinoderms [17,18] through to neuronal tasks such as neuronal differentiation [19] and neurotransmitter signalling [20] in mammalian cells. Subsequent studies have revealed regulation of TPCs by the endo-lysosomal lipid phosphatidylinositol 3,5 bisphosphate (PI(3,5)P_2_) [21,22]. However, not all studies concur, with several reports failing to demonstrate Ca^2+^ permeability and NAADP sensitivity of TPCs [21,23]. This may reflect the challenges associated with recording ion channels that do not reside on the cell surface and/or potential loss of accessory factors required for NAADP sensitivity [[24], [25], [26], [27]]. Certainly in intact cells, measurements of cytosolic Ca^2+^, albeit less direct than electrophysiology, affirm the role of TPCs in NAADP-mediated Ca^2+^ signalling [28,29].

The luminal concentration of Ca^2+^ in lysosomes is on par with that of the better characterised ER Ca^2+^ stores; ~500 μM [30]. However, lysosomes occupy a much smaller volume of the cell compared to the ER. This feature likely renders lysosomes better suited for generating localized Ca^2+^ signals. During signalling, local Ca^2+^ signals evoked by NAADP are thought to be amplified by Ca^2+^ release channels on the ER resulting in global Ca^2+^ signals that pervade the cell [31,32]. Such chatter during ‘evoked mode’ is probably underpinned by the process of Ca^2+^-induced Ca^2+^ release and facilitated by membrane contact sites between the organelles [33,34]. But growing evidence indicates that local Ca^2+^ signals act in their own – in ‘constitutive mode’ - to regulate cellular processes. Key here is the TPC interactome which reveals association with a number of proteins involved in membrane fusion, trafficking and organisation [26,35]. Such associations likely underpin roles for TPCs in regulating autophagy [36], endo-lysosomal morphology [26,37], retrograde transport from endosomes to the Golgi [38] and membrane contact site formation between late endosomes and the ER [39].

The pharmacology of TPCs is in its infancy. Indirect blockers include Ned-19 which is an NAADP antagonist discovered through ligand-based virtual screening [40]. Early work prior to the emergence of TPCs as NAADP targets, identified several L-type voltage-gated Ca^2+^ channel modulators as inhibitors of NAADP-mediated Ca^2+^ release [41]. These blockers did not affect NAADP binding suggesting a remote site of action likely on the target channel [42]. Consistent with this, recent docking analyses have identified a possible binding site within the TPC pore for L-type Ca^2+^ channel blockers [6]. This site is also predicted to accommodate local anaesthetics (Na^+^ channel blockers) which like Ca^2+^ channel blockers demonstrably inhibit endogenous NAADP-evoked Ca^2+^ signals and Ca^2+^ signals evoked by recombinant TPCs [6]. TPCs thus possess a ‘loose’ pharmacology relative to Ca^2+^ and Na^+^ channels. This suggests that core structural determinants underlying drug block were probably in place in a primordial two-domain channel early in the evolution of the voltage-gate ion channel superfamily. This is prior to the duplication events that led to extant four domain Ca^2+^ and Na^+^ channels and their subsequent divergence allowing them to distinguish Ca^2+^ and Na^+^ channel blockers [6].

**Fig. 1:**
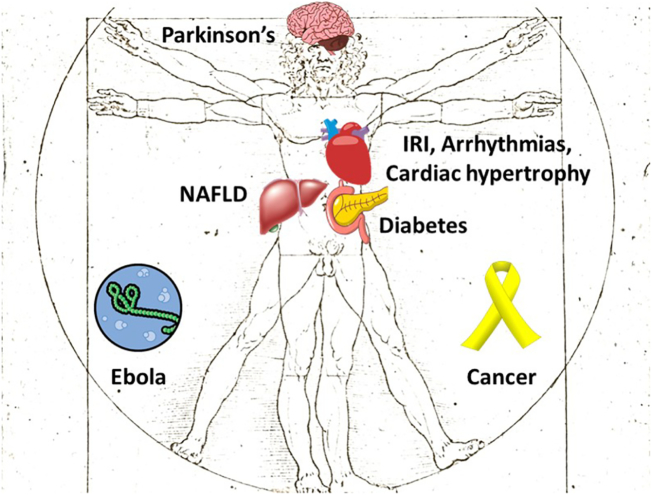
TPCs and disease. Schematic summarizing diseases associated with TPC dysfunction.

In this contribution, we review how chemical and molecular strategies are converging to uncover roles for TPCs in disease. These diseases affect a number of organs such as the brain, liver, heart and the pancreas. They include cancer and Ebola infection and possibly others (Fig. 1).

## 2 Parkinson's disease

Parkinson's disease is a common and disabling neurodegenerative disorder, characterised by a progressive decline in the control of voluntary movement. The neuropathological hallmark of Parkinson's is the selective death of dopaminergic neurons in the substantia nigra pars compacta and, in the surviving neurons, an accumulation of α-synuclein aggregates [43]. For many, the cause of the disease is unknown, however in around 10–15% of cases, it is associated with genetic mutations. TPCs have been implicated in Parkinson's mediated by mutation in the *LRRK2* gene which represents the most common cause of familial disease.

*LRRK2* encodes a large multi-domain enzyme and the prevalent G2019S mutation in Parkinson's affects its kinase domain enhancing catalytic activity [44]. It is unclear how this gain-of-function disrupts cellular function, but the endo-lysosomal system seems to be involved [45]. Indeed, several studies have shown that LRRK2 interacts with proteins on the endo-lysosomal compartment including TPCs [46] and several members of the Rab family [47] with which TPCs interact [26].

Hilfiker and colleagues identified autophagic defects in cells overexpressing wild-type and mutant (G2019S) LRRK2 [46]. These autophagic defects could be mimicked by treating cells with cell permeable NAADP and reversed through Ca^2+^ chelation, NAADP antagonism (with Ned-19) or by overexpressing a non-conducting TPC2 pore mutant [10,48]. This suggests TPC2 activity is enhanced by LRRK2. Whether LRRK2 directly phosphorylates TPCs is not known. Coupled with a recent study demonstrating that LRRK2 also associates with the voltage-gated Ca^2+^ channel CaV 2.1 and increases channel activity [49], hints that LRRK2 might have a common, hyper-activating action on Ca^2+^ channels.

LRRK2/TPC action was also studied in fibroblasts derived from Parkinson's patients with the G2019S LRRK2 mutation [37]. Using various markers, compartments of the endo-lysosomal system were demonstrably enlarged and clustered in LRRK2-Parkinson's fibroblasts relative to age matched controls. This was confirmed by other studies in neuronal cultures overexpressing mutant LRRK2 [50]. Morphology defects could be reversed by pharmacological inhibition of TPC regulators (NAADP, PI(3,5)P_2_ and the trafficking GTPase, Rab7) and molecular silencing of TPC2 (but not TPC1) [37]. Additionally, Ca^2+^ signals evoked by NAADP were enhanced in *LRRK2*- in Parkinson's. Thus, these results further support the hypothesis that pathological LRRK2 enhances TPC2 activity to cause dysfunction.

Although patients carrying mutations in LRRK2 present with a phenotype clinically and pathologically indistinguishable to idiopathic Parkinson's, it is possible that LRRK2 studies might not apply to the whole Parkinson's population. Interestingly, a recent transcriptomic meta-analysis of blood samples from sporadic Parkinson's patients found that *TPCN2* was one of the top 20 genes with altered expression [51]. Additionally, endo-lysosomal morphology was disrupted and total lysosomal Ca^2+^ levels were reduced in fibroblasts from in Parkinson's patients with a mutation in *GBA1* [52]. This gene encodes a lysosomal hydrolase and underlies the lysosomal storage disorder, Gaucher's disease. Mutations are also associated with an up to 20-fold increased risk of developing Parkinson's [53]. It is possible that lysosomal Ca^2+^ levels were depleted in GBA1-Parkinson's due to excessive TPC2 activity, however this has yet to be established. Intriguingly, a recent study demonstrated that Parkinson's is associated with multiple genes underlying other lysosomal storage diseases whereby almost half of the cohort analysed had 1 or more putative damaging mutations [54]. That lysosomal Ca^2+^, including NAADP-evoked signals [55], is disrupted in a quintessential lysosomal storage disorder (Niemann-Pick Disease Type C) identifies much scope for investigating TPC function in in Parkinson's.

In summary, pathogenic LRRK2 increases TPC2 functionality likely through a direct interaction, to disrupt autophagy and endo-lysosomal morphology. Furthermore, it is possible that TPC2 dysfunction features in other forms of Parkinson's.

## 3 Non-alcoholic fatty liver disease

Non-alcoholic fatty liver disease (NAFLD) is the most common chronic disorder of the liver characterised by fat accumulation and fibrosis. NAADP is detectable in hepatocytes [56], liver homogenates bind NAADP [7] and expression of both TPC1 and TPC2 protein has been validated in the liver using knock-out samples [35,57]. Grimm et al. analysed TPC2 knock-out mice and reported several phenotypes consistent with NAFLD [35].

In mice fed with a Western style high cholesterol diet, the livers from the transgenic animals were discoloured and weighed more than those from wild type mice [35]. Liver cholesterol levels were also increased upon TPC2 knockout. This was associated with prevalent lipid droplets and fibrosis in the liver. The presence of gall stones was also noted in the knockout animals. Additionally, circulating levels of cholesterol and liver enzymes were increased, all pointing to cholesterol overload and liver damage. Consistent with this, synthetic enzymes for cholesterol ester synthesis were transcriptionally upregulated whereas those for cholesterol synthesis were down regulated. Taken together, these data point to a major role for TPC2 in cholesterol handling [35].

Mechanistic studies in mouse embryonic fibroblasts suggest that TPC2 knockout disturbs trafficking of LDL receptors [35]. LDL receptors mediate uptake of cholesterol through endocytosis, initiating delivery of cholesterol to the lysosome and beyond through both vesicular and non-vesicular means [58]. In TPC2 null cells, LDL appeared to accumulate in punctate structures [35]. Similar accumulation was seen with EGF which like LDL is internalised *via* receptor-mediated endocytosis and delivered to the lysosome [59]. Pulse chase experiments with EGF suggest a slowing of degradation [35]. However, neither the pH nor the proteolytic activity of lysosomes was altered pointing to compromised transport through the endocytic system. Indeed, EGF accumulated in Rab7 (late endosome/lysosome) positive structures leading the authors to speculate that a Ca^2+^-dependent block in fusion between late endosomes and lysosomes was the underlying cause. Consistent with this, the effects of TPC2 knockout on EGF distribution could be pheno-copied by buffering local Ca^2+^ changes with BAPTA and rescued by a wild-type TPC2 but not a non-conducting pore mutant [10,48]. Whether a similar mechanism is responsible for accumulation of LDL remains to be established but other studies suggest a modest slowing of PDGF receptor degradation upon PDGF stimulation in TPC2 (but not TPC1) knockout cells [60]. Again, this appears to be independent of changes in lysosomal pH [60]. Further work however is required to identify the exact ‘lesion’ within the endo-lysosomal system that TPC2 deletion causes given the accumulation of EGF in LAMP1 (predominantly lysosomal) as well as Rab7-positive structures [35].

In summary, de-regulated trafficking of receptors upon TPC2 knockout probably through disrupted local Ca^2+^ signalling provides a likely mechanism for liver dysfunction in NAFLD.

## 4 Ebola virus disease

The Ebola virus causes an often fatal haemorrhagic fever in humans exemplified by the 2014 outbreak in West Africa. Work by Davey and colleagues identified TPCs as potential drug targets for combatting Ebola infection [61].

Many viruses highjack the endocytic system to enter cells [62]. For Ebola, cleavage of the Ebolavirus glycoprotein by cysteine proteases within late endosomes/lysosomes is an essential step triggering binding to NPC1, subsequent fusion and escape of the viral core into the cytoplasm [63]. Sakurai et al. found that knock down or knockout of either TPC1 or TPC2 prevented Ebola infection *in vitro* [61]. Over-expression of non-conducting TPC2 pore mutant [10,48] also inhibited infection. So too did a number of voltage-gated Ca^2+^ channel blockers and Ned-19. The former included tetrandrine a plant alkaloid derived from a Chinese herb that is often used in traditional medicine [64]. Tetrandrine was particularly potent with an affinity in the nanomolar range. Importantly, tetrandrine proved efficacious *in vivo* in a mouse model of Ebola infection [61].

Mechanistically, tetrandrine blocked NAADP- and PI(3,5)P_2_-stimulated currents through TPC1 and TPC2, and NAADP-evoked Ca^2+^ signals live cells [61]. It also resulted in accumulation of virus particles in TPC2- and NPC1-positive compartments. This was associated with depletion of virus particles in TPC2-positive but NPC1-negative compartments. This led the authors to speculate that the virus leaves through the TPC2-only compartment because of the correlation between reduced presence of virus in this compartment and reduced infection [61]. However, one could argue that blocking TPCs would result in accumulation of virus in the compartment that it normally exits from i.e. the TPC2/NPC1-positive compartment. Simmons et al. found that viruses appeared to exit exclusively from NPC1-positive compartments and found no evidence for compartments that were TPC2-positive but NPC-negative [65]. One potential caveat in both studies [61,65] is the use of overexpressed TPC2 for assessing colocalization given the effect of TPC2 on perturbing endo-lysosomal morphology [26]. Unexplained at this point, is the mechanistic requirement for TPC1 in Ebola infection given its likely more proximal (endosomal) targeting within the endo-lysosomal system relative to TPC2 (lysosomal) [39,66]. The precise subcellular localisation of endogenous channels however is still unclear due to lack of appropriate antibodies and the ‘fluid’ nature of the endo-lysosomal system whereby different organelles are undergoing constant maturation and fusion. Further work is required to determine exactly how TPCs allow virus escape.

In summary, molecular and chemical targeting of TPCs provides a novel strategy for combatting Ebola uncovering a role for TPCs in viral entry probably through regulation of Ca^2+^-dependent fusion events.

## 5 Cancer

Cancer needs little introduction. It is a pervasive problem. Many cellular processes are subverted during carcinogenesis including the process of cell migration (underlying metastasis) and angiogenesis (underlying tumour vascularisation). TPC expression in cancer was originally characterised in SKBR3 (human breast cancer) and PC12 (rat pheochromocytoma) cells where TPC1 transcript levels were reported to be ~3–8 fold higher than TPC2 [8]. TPCs are also expressed in a number of other breast cancer lines [67].

**Fig. 2:**
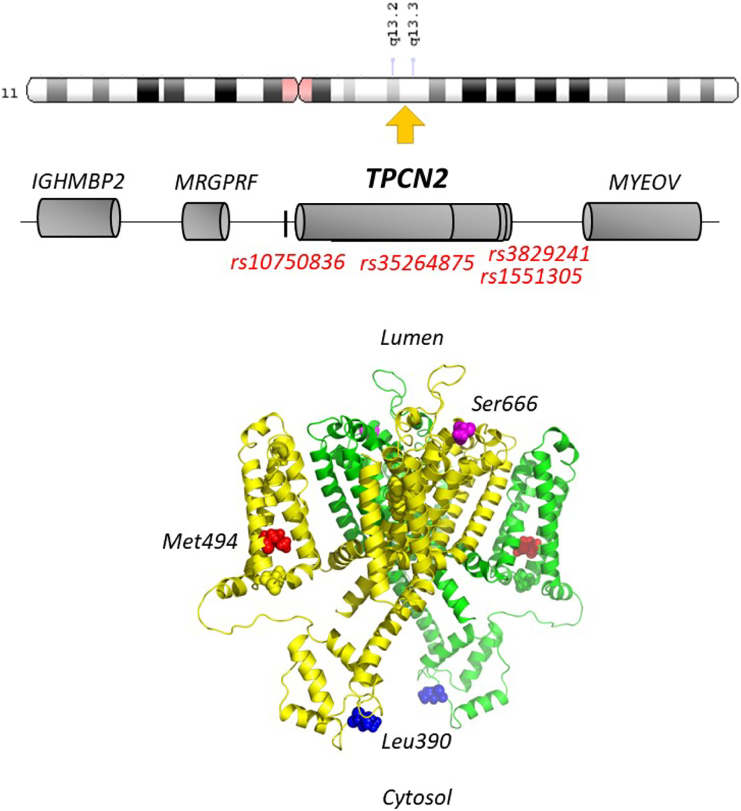
Structural features of TPC2. Schematic depicting *TPCN2* and flanking genes (centre) on chromosome 11 (top). Approximate positions of single nucleotide variants are marked. A structural model of human TPC2 (bottom) highlighting the position of a pigmentation-linked human variant (red), a variant linked to diabetic traits in rat TPC2 (blue) and a proposed PKA phosphorylation site (magenta).

In humans, the genes for TPC1 and TPC2 are found on chromosome 12 (*TPCN1*, NCBI Gene ID: 53373) and 11 (*TPCN2*, NCBI Gene ID: 219931), respectively. The gene encoding TPC3, which is surprisingly absent from select mammals including the mouse, is a pseudogene in humans but likely functional in closely related primates [13,68]. The chromosomal region harbouring *TPCN2* (11q13.2, Fig. 2) is often amplified in cancer and also contains the cyclin gene which is an established driver of oncogenesis [69]. Interestingly, increased expression of TPC2 and a number of other nearby genes in a proposed ‘cassette’ has been observed in oral squamous cell carcinoma cell lines and primary tumours [70,71]. Amongst the cassette, which may comprise two ‘cores’ [72], is TMEM16A which encodes a Ca^2+^-activated Cl^−^ channel potentially pointing to this protein as an effector of de-regulated Ca^2+^ signals in cancer.

Increased expression of both TPC1 and TPC2 has been noted in several cell lines derived from bladder, blood and liver cancer relative to a malignant breast cancer line [73]. This was particularly striking for TPC1 which was elevated up to ~25-fold. Knockdown of TPC1 or TPC2 and antagonising NAADP action with, Ned-19 or inhibiting channel activity of TPCs with tetrandrine, were all found to inhibit migration of cancer lines. This points to TPCs as potential therapeutic targets in cancer [74] although increased expression of TPC2 is correlated with increased survival in bladder cancer [75]. Mechanistically, these manoeuvres were associated with enlarged lysosomal structures. Enlargement of endo-lysosomal structures by molecular or chemical interference of TPCs/NAADP has been reported in primary cultured fibroblasts although in this case, the effects were specific to TPC1 [39].

Inhibiting TPCs was also associated with reduced adhesion and reduced formation of the leading edge in cancer cell lines [73]. These findings translated to reduced metastatic tumour formation in an *in vivo* mouse model of metastasis although these experiments used a mammary cell reporter line. In this context, it is worth noting that the role of Ca^2+^ signalling from acidic organelles in the migration neural crest cells [76]. Neural crest cells are an embryonic population of cells born in the neural plate and which migrate through the embryo during early development [77]. Migration of these cells is often likened to metastasis. Knocking down an identified animal Ca^2+^-H^+^ exchanger in *Xenopus*, which is likely responsible for filling acidic organelles with Ca^2+^, was found to disrupt neural crest cell migration *in vivo* [78]. *In vitro*, this was also associated with reduced adhesion as evidenced by reduced dispersion and reduced focal adhesions. This could be recapitulated in wild type cells by the fast Ca^2+^ buffer, BAPTA but not the slower buffer, EGTA. These data point to local Ca^2+^ signals regulating the adhesion machinery [78]. Indeed, identified defects in integrin trafficking in mammalian cancer lines upon TPC knockdown was also ascribed to de-regulated local Ca^2+^ fluxes [73]. This is similar to the proposed mechanism underlying defective trafficking of LDL/EGF receptors upon TPC2 knockout [35]. Although it should be noted that trafficked cargo in TPC compromised cells accumulated in distinct organelles; early endosomes (integrins) [73] *vs* lysosomes/late endosomes (EGF receptors) [35].

Global Ca^2+^ signals deriving from acidic Ca^2+^ stores might also contribute to cancer. NAADP signalling has previously been implicated in agonist-evoked Ca^2+^ signalling in endothelial cells in response to cues such acetylcholine [79] and histamine [80]. Coupling of VEGF to NAADP/TPC signalling [81] is relevant in the context of angiogenesis - the process whereby endothelial cells give rise to new blood vessels. Here, endothelial cells breach the basement membrane and undergo migration and proliferation to form new vessel structures. This process is essential not only during development but also during cancer, where vascularisation feeds solid tumours. Work by Favia et al. found that TPCs were required for several aspects of VEGF-induced angiogenesis including migration of endothelial cells and tube formation *in vitro* [81]. Importantly, *in vivo* Matrigel assays showed that vascularisation by VEGF was inhibited by Ned-19 and in TPC2 but not in TPC1 knockout mice. TPC2 knockout mice however appeared to develop normally suggesting that compensatory changes maintain angiogenesis during development of these animals. Nevertheless, these studies highlight a role for TPCs in a process crucial for cancer progression.

Melanoma is a cancer that derives from pigmented melanocytes. A link between pigmentation and TPCs has been long appreciated through GWAS which correlated two non-synonymous variants in TPC2 with hair colour [82]. Overexpression of TPC2 (but not TPC1) induces pigmentation defects in *Xenopus* oocytes in a manner that requires interactivity with members of the Rab family of trafficking proteins [26]. And, a recent study suggested that both pigmentation variants in TPC2 result in a gain of channel function through either conformational changes within the pore (rs35264875, M484L) or reduced inhibition by mTOR (rs3829241, G734E) (Fig. 2) [83]. Collectively, these data link the level of TPC2 activity to pigmentation. But exactly how TPC2 regulates pigmentation is not clear at present. TPC2 localises to pigment bearing melanosomes (notably an acidic Ca^2+^ store) to potentially regulate melanin production through changes in luminal pH [84,85]. However, despite strong links between pigmentation and cancer there is still only suggestive genetic evidence linking TPC2 variants with melanoma which might be sex-specific [86].

In summary, cancer likely features changes in TPC expression and defects in both local and global TPC-mediated Ca^2+^ signalling. TPCs seem to be required for cellular processes that underpin cancer cell survival. And thus present themselves as viable therapeutic targets.

## 6 Cardiac dysfunction

The actions of NAADP in the heart have been long appreciated from early radioligand binding and flux assays using cardiac microsomes [87] through to more recent imaging studies resolving NAADP-evoked Ca^2+^ signals in individual live cells [88]. TPCs are demonstrably expressed in the heart possibly in a sex-specific manner whereby levels in female hearts were greater than male hearts [89]. TPCs have been implicated in ischemia reperfusion injury, arrhythmias and cardiac hypertrophy.

Interrupting blood supply to the heart causes ischemia and tissue injury. This can occur during myocardial infarction. Reperfusion is essential to restore oxygen and nutrients. But this process in itself exacerbates damage through opening of the mitochondrial transition pore. Ca^2+^ has long been considered an upstream culprit in ischemia-reperfusion injury but the underlying mechanisms are not well understood.

TPC1 and TPC2 levels are reportedly higher in left ventricular samples from ischemic as well as dilated hearts from patients suffering from heart failure [90]. Davidson et al. found that TPC1 knock-out mice showed reduced cardiac damage in an *in vivo* model of ischemia-reperfusion injury [91]. This effect was pheno-copied by blocking NAADP action with Ned-K, a novel Ned-19 analogue. *In vitro*, Ca^2+^ oscillations, and subsequent cell death of cardiomyocytes upon re-oxygenation following ischemia were inhibited by Ned-K and the lysosomotropic agent, GPN. The principle Ca^2+^ release channel in cardiomyocytes is the type 2 ryanodine receptor and previous studies have implicated this protein in reperfusion-induced Ca^2+^ oscillations [92]. This led to a model whereby activation of TPCs likely triggers Ca^2+^ release from the sarcoplasmic reticulum which is ultimately received by mitochondria resulting in MTP opening [91]. Knockdown of TPCs exacerbated cell death of cardiomyocytes during extended ischemia (no reperfusion) pointing to possible context-specific roles for TPCs in regulating cardiac viability [89].

How TPCs are activated during reperfusion remains to be established. NAADP levels decrease upon ischemia and increase upon reperfusion [91]. However, the absolute levels upon reperfusion are no higher than prior to ischemia. Thus, changes in NAADP levels *per se* are unlikely to trigger Ca^2+^ release through TPCs. One possibility is that TPCs become sensitised to NAADP during re-perfusion perhaps in response to oxidative stress, a known determinant of ischemia-reperfusion injury. Indeed, plant TPCs are reportedly responsive to oxidants [93].

Interestingly, extracellular NAADP appears to paradoxically protect hearts against ischemic injury [94,95]. Such effects were reversed by an antagonist of P2Y11 receptors or Ned-19 suggesting NAADP acts on the cell surface or intracellularly upon transport, respectively. Further mechanistic studies are warranted to relate these effects to TPCs.

Adrenaline plays a key role in the pathophysiology of the heart. Low level stimulation of β-adrenergic receptors acutely increases contractility (classically during the ‘fight or flight’ stress response) whereas more intense stimulation induces arrhythmias. Chronic stimulation induces hypertrophy. Accumulating evidence points to NAADP as a second messenger for adrenaline in the heart [[96], [97], [98]].

Work by Guse and colleagues provided evidence that the NAADP antagonist BZ194 [99] could prevent isoprenaline-induced spontaneous diastolic Ca^2+^ transients in electrically-paced cardiomyocytes and arrhythmias *in vivo* [88]. The arrhythmic Ca^2+^ transients were also prevented by bafilomycin indicating a requirement for acidic organelles and thus pointing to TPCs. Terrar and colleagues provided direct evidence for TPC2 in the actions of adrenaline [100]. In their study, isolated hearts from a TPC2 knock-out animals were less susceptible to arrhythmias upon ventricular burst pacing compared to wild type hearts. The hypertrophic response to isoprenaline *in vivo* was also modestly reduced upon TPC2 knock-out.

Mechanistically, these blunted responses were attributed to reduced action of adrenaline on the amplitude of evoked Ca^2+^ transients, effects that could be mimicked by Ned-19 and bafilomycin [100]. Bafilomycin however did not affect the amplitude of the Ca^2+^ transients in previous work [88]. Nevertheless, these analyses suggest that adrenaline action which is traditionally attributed to cyclic AMP-mediated phosphorylation of Ca^2+^ handling proteins such as voltage-gated Ca^2+^ channels, and more controversially ryanodine receptors, may be more complex. It is tempting to speculate that cyclic AMP-dependent protein kinase phosphorylates TPCs to augment their action. Indeed, evidence for positive regulation of TPC2 by PKA has recently been provided [101]. However, the proposed phosphosite (S666) appears to be situated at the start of the sixth trans-membrane region in domain II and therefore luminal (Fig. 2). This questions its physiological relevance. Downstream of TPC activation, Ca^2+^ may enhance Ca^2+^ release from the SR possibly through promoting Ca^2+^ uptake through a CamKinase II-dependent mechanism [100].

In sum, TPCs have emerged as common mediators of cardiac dysfunction in several scenarios of heart disease through disrupting global Ca^2+^ signalling. Chemical targeting of NAADP *in vivo* has proven beneficial.

## 7 Diabetes

Diabetes is a metabolic disorder characterised by hyper-glycemia resulting from either deficient secretion of insulin or insulin action. Functional NAADP effects in the insulin secreting beta cells of the endocrine pancreas has been long known [102] where it is considered a second messenger for glucose [103] and possibly insulin [104] and GLP-1 [105]. Several lines of evidence link TPCs to diabetes.

Insulin is a key anabolic hormone that controls blood sugar by promoting glucose uptake into peripheral tissues. It is secreted by beta cells in a Ca^2+^-dependent manner in response to glucose. The prevailing view is that this is due to inhibition of ATP-sensitive K^+^ channels, membrane depolarisation and activation of voltage-gated Ca^2+^ channels. However, it is increasingly appreciated that other mechanisms likely contribute to stimulus-secretion coupling [106]. Indeed, it has long been reported that glucose-induced Ca^2+^ signals can be inhibited by interfering with NAADP [40,103]. Consistent with this, isolated beta cells from mice lacking TPC1 or TPC2 show reduced glucose-evoked Ca^2+^ signals [125]. Double knockout mice however appear unperturbed [21] although TPC expression in these animals was not characterised [28].

Importantly, whole pancreata derived from TPC1 knock-out mice secrete less insulin in response to elevated glucose [125]. Moreover, *in vivo* studies of these animals showed that blood glucose levels were modestly increased in glucose tolerance tests. Reduced insulin secretion and elevated blood glucose are both key diabetic traits. Mice lacking TPC2 also show decreased insulin secretion in response to glucose challenge *in vivo* [107] although *ex vivo* studies using whole pancreata were less clear [125]. Glucose levels in TPC2 knockout mice *in vivo* were paradoxically either slightly reduced [107] or unchanged [125]. Beta-cell specific knockout of TPC2 did not affect glucose-evoked Ca^2+^ signalling, insulin secretion or glucose tolerance [108]. One possibility to explain these discordant results is that TPC knockout is compensated for *in vivo*, a perennial concern for transgenic mice studies. Here, a recent study focusing on alpha cells of the endocrine pancreas is potentially relevant. Alpha cells secrete glucagon which has the opposite effect to insulin on blood sugar levels. Knock-out of TPC2 but less so TPC1 was shown to reduce glucagon secretion [109]. Consequently, global TPC2 knock-out at least may mask its role in glucose homeostasis *in vivo*. Clearly further studies are required to relate loss of TPCs in single cells through organs to whole animal phenotypes particularly for TPC2.

In rats, TPC2 is present in a genomic locus on chromosome 1 that mapped previously to glucose intolerance [110]. Interestingly, it was the only gene in this locus (and 1 of 47 others genome wide) whose expression levels changed in glucose intolerant rats [107]. TPC2 levels negatively correlated with fasting glucose levels. Moreover, several variants in this locus associated with TPC expression levels. These analyses suggest that TPC2 expression may be causally related to glucose perturbances [107]. Interestingly, one of the variants resulted in a non-synonymous coding change in TPC2 itself (P350L). In mouse TPC1, for which the structure was recently resolved, the corresponding residue is leucine and located between the second E and F helicies that form an E-F like hand-like domain in the linker. In plants, this region binds Ca^2+^ but in mammalian TPCs, lack of Ca^2+^ coordinating residues likely renders this region Ca^2+^-insensitive. In human TPC2, the corresponding residue is again leucine like the rat TPC2 variant (Fig. 2). The coding change is predicted to be benign but its exposed nature may affect interactions between TPC2 and regulatory proteins in the cytosol.

A variant in the human *TPCN2* gene (rs1551305) has also been implicated in diabetes. Fan et al. identified a variant within an intron toward the 3′ of the gene (Fig. 2) that associates with type 2 diabetes and reduced beta cell function [111]. It would be interesting to examine TPC2 levels in this cohort.

In sum, studies in both rodent models and humans support a role for TPCs in regulating global Ca^2+^ signals and endocrine pancreatic function. Knockout of TPCs in some but not all models recapitulates diabetic traits. And genetic studies further link TPCs to diabetes possibly through changes in TPC levels.

## 8 Other disorders

Mutation of presenilins (PS1 and PS2) underlie familial forms of Alzheimer's disease [112]. In cells lacking PS1 and PS2, lysosomal Ca^2+^ content is reduced [113] and this is associated with altered post-translational modification of TPCs (probably glycosylation) [114]. In PS1-knockout cells, NAADP-evoked Ca^2+^ release is reduced consistent with reduced store content but Ca^2+^ release by the TRPML agonist is paradoxically enhanced [115]. Intriguingly, the latter could be reversed by Ned-19 [115]. This warrants further work to better define the relationships between TPCs, TRPMLs and NAADP in Alzheimer's.

Further phenotypic analyses of TPC knockout mice have implicated TPC1 in infertility through a requirement for the acrosome reaction in sperm [116]. The Mendelian ratio of offspring was slightly affected upon TPC1 knockdown pointing to a sub-fertile phenotype. TPC2 has been implicated in skeletal muscle atrophy through proposed regulation of autophagic signalling [117]. This follows on from work demonstrating a requirement for NAADP/TPCs in autophagy [36,46]. But further corroborating evidence is required to causally link skeletal muscle function to autophagy/lysosomal function given the reported increase in lysosomal pH upon TPC2 knockout [117] in light of other studies where pH was unaffected [28,35,117]. Analyses of TPC1 and TPC2 double knockout mice suggests a role for TPCs in mature onset obesity [118]. But an effect on body weight was not noted in a previous analyses of TPC2 knockout mice even when fed on a cholesterol rich diet which induced major liver dysfunction [35].

Interestingly, a recent GWAS linked TPC2 to systemic lupus erythematosus [119], a chronic autoimmune disease. A variant (rs10750836) located upstream of the TPCN2 gene (Fig. 2) was found to correlate with reduced TPC2 expression levels in B cells from patients [119]. This effect was supported by reporter expression analyses. Functional studies of B cells upon TPC2 knockout/inhibition is eagerly awaited not least due to the essential role for lysosomal compartments in antigen processing.

In sum, TPCs might be involved in a number of additional disorders affecting the brain, reproductive system, skeletal muscle, adipose tissue and the immune system. But further supporting evidence is required.

## 9 Closing remarks

As discussed, NAADP antagonists have proven beneficial in correcting disease phenotypes in cellular and animal models of Parkinson's (Ned-K), cancer (Ned-19) and heart arrhythmia (BZ194), often mimicking the effects of TPC knockdown/knockout. They, thus present themselves as lead drugs for combatting TPC dysfunction. But because the effects of NAADP on TPCs is likely indirect, a clear mechanism of action is difficult to ascertain as NAADP-binding proteins continue to evade molecular identification.

An alternative (and complementary) approach to manipulate this pathway is to directly target TPCs. Success with tetrandrine in combatting Ebola infection is most encouraging in this respect although its pleiotropic actions should not be ignored [64]. Indeed, chemical targeting of Ca^2+^ channels is not without its challenges. Nevertheless, successful drugging of voltage-gated Ca^2+^ channels (with for example dihydropyridines) and ryanodine receptors (with dantrolene) in hypertension and malignant hyperthermia offer hope. In this context, emerging structures of TPCs [5,[120], [121], [122],^126^] should aid in the rational design of TPC blockers.

Finally, with growing evidence linking disorders of the pancreas and liver with loss of TPC function should focus attention on strategies to boost TPC activity. It is intriguing that tolbutamide, a diabetic drug which targets kATP channels to stimulate insulin secretion appears to require TPC2 for its activity [125]. Administration of NAADP itself is reportedly beneficial in diabetes [123] but exactly how NAADP enters cells requires work. A major hurdle at present is the lack of TPC agonists. Here, TRP mucolipins which like TPCs localise to the endo-lysosomal system to (de-)regulate Ca^2+^ dependent output, lead the way with the availability of both activators and inhibitors [124].

No doubt future advances will offer us new approaches to target TPCs in disease.

## Transparency document

Transparency document. Image 1
